# Supplementary material for: Thiamine and Biotin: Relevance in the Production of Volatile and Non-Volatile Compounds during Saccharomyces cerevisiae Alcoholic Fermentation in Synthetic Grape Must
Source: Foods. 2023 Feb 24;12(5):972. doi: 10.3390/foods12050972 (PMC10000645; doi:10.3390/foods12050972)
Supplement: Supplementary file 1 [file foods-12-00972-s001.zip › foods-2190344-supplementary.pdf]

Supplementary Materials

Table S1. Impact of both biotin and thiamine on *Saccharomyces cerevisiae* growth and fermentation kinetics in synthetic wine.

		Initial Thiamine Concentration ($\mu g.L^{-1}$)		
Initial Biotin		0	50	250
0	μmax (h^{-1})[1]	0.46 ± 0.07	0.41 ± 0.06	0.43 ± 0.08
	G (h)[2]	1.5 ± 0.2	1.7 ± 0.2	1.7 ± 0.0
	y_{max} ($cells.mL^{-1}$)[3]	$1.02.10^8 \pm 1.02.10^7$	$7.05.10^7 \pm 8.15.10^6$	$6.45.10^7 \pm 5.25.10^6$
	t_{AF} (h)[4]	133.6 ± 0.0	149.6 ± 13.9	133.6 ± 0.0
	rCO2max ($gCO2.200\ mL^{-1}.h^{-1}$)[5]	0.19 ± 0.02	0.15 ± 0.02	0.20 ± 0.07
0.5	μ_{max}	0.43 ± 0.03	0.43 ± 0.01	0.43 ± 0.02
	G	1.6 ± 0.1	1.6 ± 0.0	1.6 ± 0.1
	y_{max}	$3.31.10^8 \pm 2.67.10^7$	$2.30.10^8 \pm 5.06.10^7$	$1.67.10^8 \pm 7.78.10^6$
	t_{AF}	133.6 ± 24.0	133.6 ± 0.0	93.4 ± 14.0
	r_{CO2max}	0.18 ± 0.04	0.18 ± 0.02	0.26 ± 0.05
3	μ_{max}	0.42 ± 0.00	0.54 ± 0.01	0.48 ± 0.02
	G	1.6 ± 0.0	1.3 ± 0.0	1.5 ± 0.1
	y_{max}	$1.62.10^8 \pm 2.93.10^7$	$1.59.10^8 \pm 1.58.10^7$	$1.34.10^8 \pm 9.68.10^6$
	t_{AF}	141.6 ± 27.7	133.6 ± 24.0	93.4 ± 14.0
	r_{CO2max}	0.16 ± 0.03	0.17 ± 0.01	0.24 ± 0.01

[1]μ_{max}: maximal specific growth rate; [2]G: generation time; [3]y_{max}: maximal population; [4]t_{AF}: durationof the alcoholic fermentation; [5]r_{CO2max}: maximal CO2 production rate.

Table S2. *Saccharomyces cerevisiae* impacted metabolic pathways in high and low biotin conditions.

High Biotin Metabolome	
**Metabolic Pathway**	**Number of Annotated Metabolites**
Carbohydrate metabolism	**2**
2-Oxocarboxylic acid metabolism	1
Propanoate metabolism	1
Amino acid metabolism	**2**
β-alanine metabolism	1
Valine, leucine and isoleucine biosynthesis	1
Biosynthesis of secondary metabolites	***1***
Lipid metabolism	***1***
Ether lipid metabolism	1
Unclassified metabolic pathway	***4***

Low Biotin Metabolome	
**Metabolic Pathway**	**Number of Annotated Metabolites**
Carbohydrate metabolism	***1***
Fructose and mannose metabolism	1
Metabolism of cofactors and vitamins	***1***
Riboflavin metabolism	1
Unclassified metabolic pathway	***1***

Table S3. *Saccharomyces cerevisiae* impacted metabolic pathways in high and low thiamine conditions.

High Thiamine Metabolome	
**Metabolic Pathway**	**Number of Annotated Metabolites**
Biosynthesis of secondary metabolites	***20***
Biosynthesis of various plant secondary metabolites	2
Carbohydrate metabolism	***51***
2-Oxocarboxylic acid metabolism	10
C5-branched dibasic acid metabolism	7
Pyruvate metabolism	6
Propanoate metabolism	5
Ascorbate and aldarate metabolism	4
Butanoate metabolism	4
Fructose and mannose metabolism	4
Glyoxylate and dicarboxylate metabolism	4
Pentose and glucuronate interconversions	3
Citrate cycle (TCA cycle)	1
Phosphonate and phosphinate metabolism	1
Unspecified carbon metabolism	2
Amino acid metabolism	***53***
Biosynthesis of amino acids	10
Phenylalanine metabolism	8
Valine, leucine & isoleucine biosynthesis	8
Tyrosine metabolism	6
Lysine degradation	5
D-amino acid metabolism	3
Valine, leucine and isoleucine degradation	3
Cyanoamino acid metabolism	2
Cysteine and methionine metabolism	2
Glutathione metabolism	2
Alanine, aspartate and glutamate metabolism	1
Glycine, serine and threonine metabolism	1
Lysine biosynthesis	1
Phenylalanine, tyrosine and tryptophan biosynthesis	1
Lipid metabolism	***5***
Ether lipid metabolism	1
Fatty acid biosynthesis	1
Fatty acid degradation	1
Fatty acid elongation	1
Glycerolipid metabolism	1
Metabolism of cofactors and vitamins	***12***
Nicotinate and nicotinamide metabolism	4
Pantothenate and CoA biosynthesis	3
Biosynthesis of cofactors	2
Porphyrin metabolism	1
Thiamine metabolism	1
Ubiquinone and other terpenoid-quinone	1

biosynthesis	
Nucleotide metabolism	***2***
Purine metabolism	1
Pyrimidine metabolism	1
Energy metabolism	***4***
Methane metabolism	2
Oxidative phosphorylation	1
Sulfur metabolism	1
ABC transporters	***3***
Aminoacyl-tRNA biosynthesis	***2***
Unclassified metabolic pathway	***49***

Low Thiamine Metabolome

**Metabolic Pathway**	**Number of Annotated Metabolites**
Carbohydrate metabolism	***1***
Butanoate metabolism	1
Metabolism of cofactors and vitamins	***1***
Riboflavin metabolism	1
Unclassified metabolic pathway	1

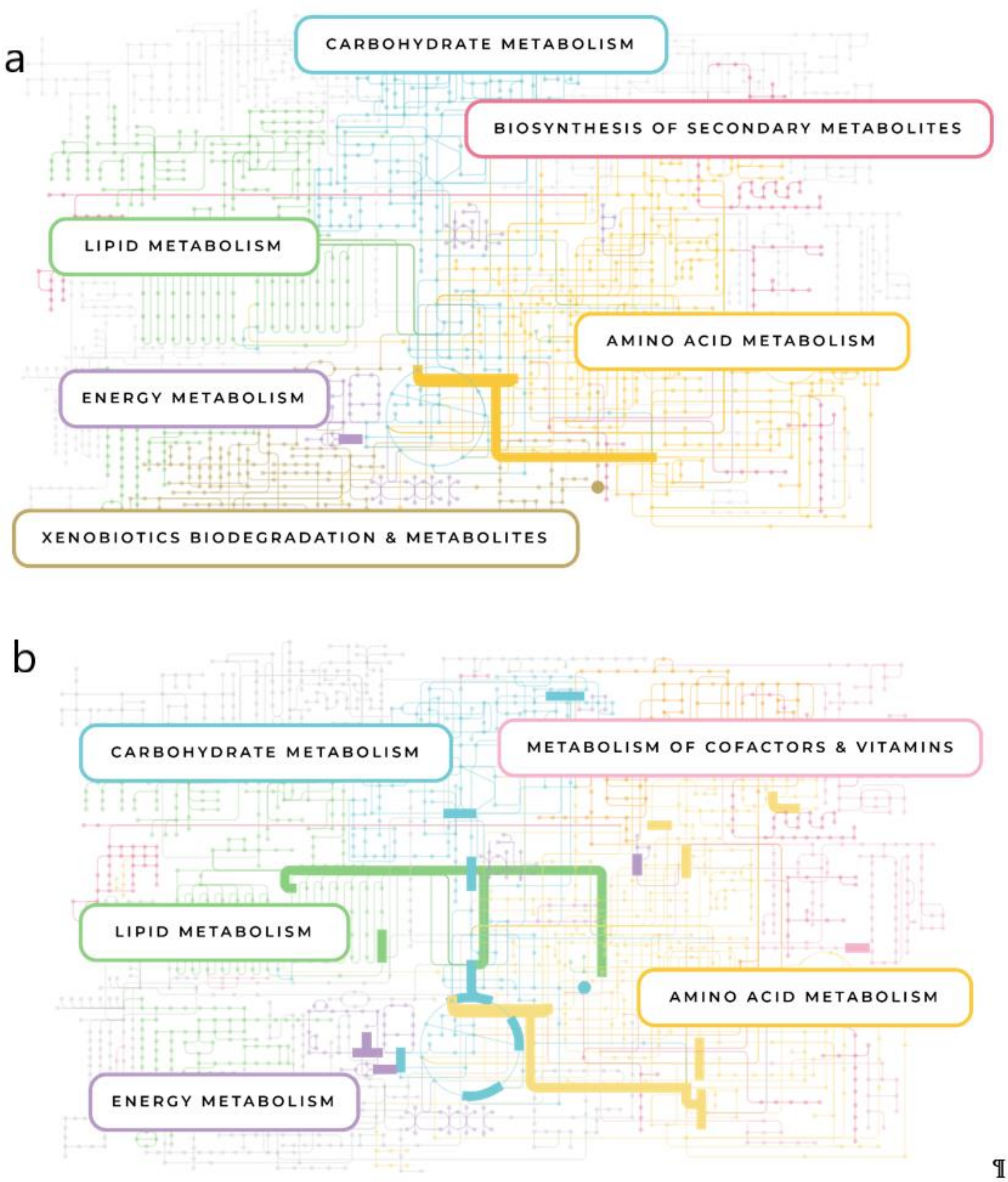

¶

Figure S1. Metabolic impact of biotin (a.) and thiamine (b.) on *S. cerevisiae*. Thicker strokes highlight the reactions and compounds in which vitamin significantly impacted metabolites were involved and found.
